# Yield and Selectivity Improvement in the Synthesis of Carbonated Linseed Oil by Catalytic Conversion of Carbon Dioxide

**DOI:** 10.3390/polym13060852

**Published:** 2021-03-10

**Authors:** David Alejandro González Martínez, Enrique Vigueras Santiago, Susana Hernández López

**Affiliations:** Laboratorio de Investigación y Desarrollo de Materiales Avanzados, Facultad de Química, Universidad Autónoma del Estado de México, Campus Rosedal, Toluca 50200, Mexico; dgonzalezmartinez31@gmail.com (D.A.G.M.); eviguerass@uaemex.mx (E.V.S.)

**Keywords:** carbonation reaction, selectivity optimization, carbonated epoxidized linseed oil, non-isocyanate polyurethane

## Abstract

Carbonation of epoxidized linseed oil (CELO) containing five-membered cyclic carbonate (CC5) groups has been optimized to 95% by reacting epoxidized linseed oil (ELO) with carbon dioxide (CO_2_) and tetrabutylammonium bromide (TBAB) as catalysts. The effect of reaction variables (temperature, CO_2_ pressure, and catalyst concentration) on the reaction parameters (conversion, carbonation and selectivity) in an autoclave system was investigated. The reactions were monitored, and the products were characterized by Fourier Transform Infrared Spectroscopy (FT-IR), carbon-13 nuclear magnetic resonance (^13^C-NMR) and proton nuclear magnetic resonance (^1^H-NMR) spectroscopies. The results showed that when carrying out the reaction at high temperature (from 90 °C to 120 °C) and CO_2_ pressure (60–120 psi), the reaction’s conversion improves; however, the selectivity of the reaction decreases due to the promotion of side reactions. Regarding the catalyst, increasing the TBAB concentration from 2.0 to 5.0 *w*/*w*% favors selectivity. The presence of a secondary mechanism is based on the formation of a carboxylate ion, which was formed due to the interaction of CO_2_ with the catalyst and was demonstrated through ^13^C-NMR and FT-IR. The combination of these factors makes it possible to obtain the largest conversion (96%), carbonation (95%), and selectivity (99%) values reported until now, which are obtained at low temperature (90 °C), low pressure (60 psi) and high catalyst concentration (5.0% TBAB).

## 1. Introduction

Obtaining cyclic carbonates (CCs) has received significant attention because CCs have attractive properties, such as low toxicity, high solubility, and boiling points, can be used as solvents, and have high reactivity with amines [1,2]. Although 5-membered cyclic carbonates (CC5) are less reactive than 6-, 7-, and 8-element cyclic carbonates (CC6, CC7, and CC8), respectively, the synthesis of CC5 has been more studied because CC5 synthesis does not involve the use of toxic precursors such as CS_2_, phosgene, and ethyl chloroformate, among other environmentally harmful solvents [3,4]. The most common synthesis method of CC5 remains the insertion of carbon dioxide (CO_2_) in cyclic ethers because it is considered a safe process, and the atom economy is close to 100% [4]. This synthesis route has been extensively studied with small molecules such as ethylene and propylene oxide [2,5], while long-chain molecules, such as vegetable oils (VOs), have been less studied. Carbonated vegetable oils (CVOs) have various applications as solvents, lubricants, additives, plasticizers, and monomers for the formation of polymers [6,7,8,9]. Currently, the main application is in the synthesis of non-isocyanate polyurethanes (NIPUs) through the aminolysis reaction of CVO because NIPU does not require highly toxic materials such as isocyanates [10,11,12,13,14,15,16].

**Scheme 1:**
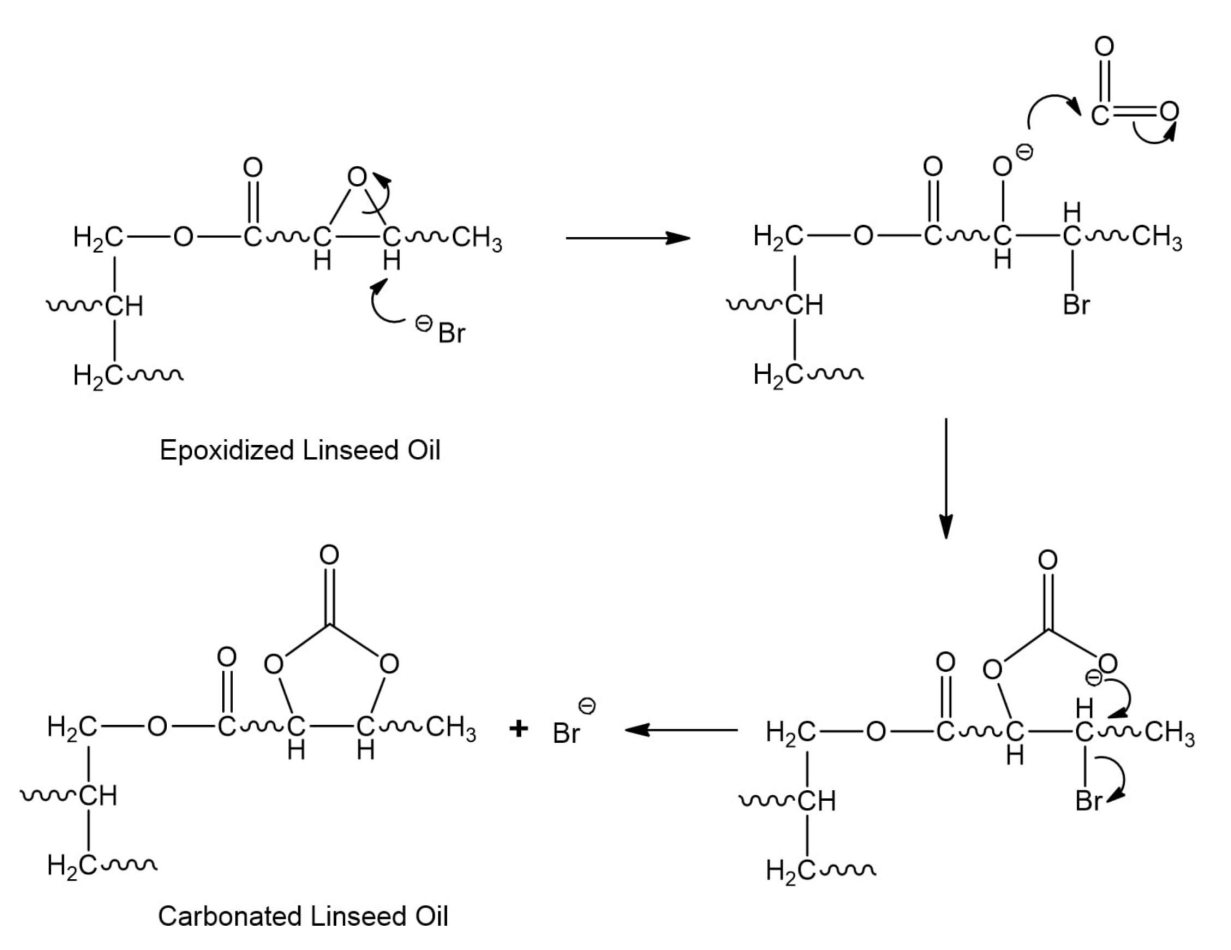
Cycloaddition reaction of carbon dioxide (CO_2_) into oxirane rings [18].

**Scheme 2:**
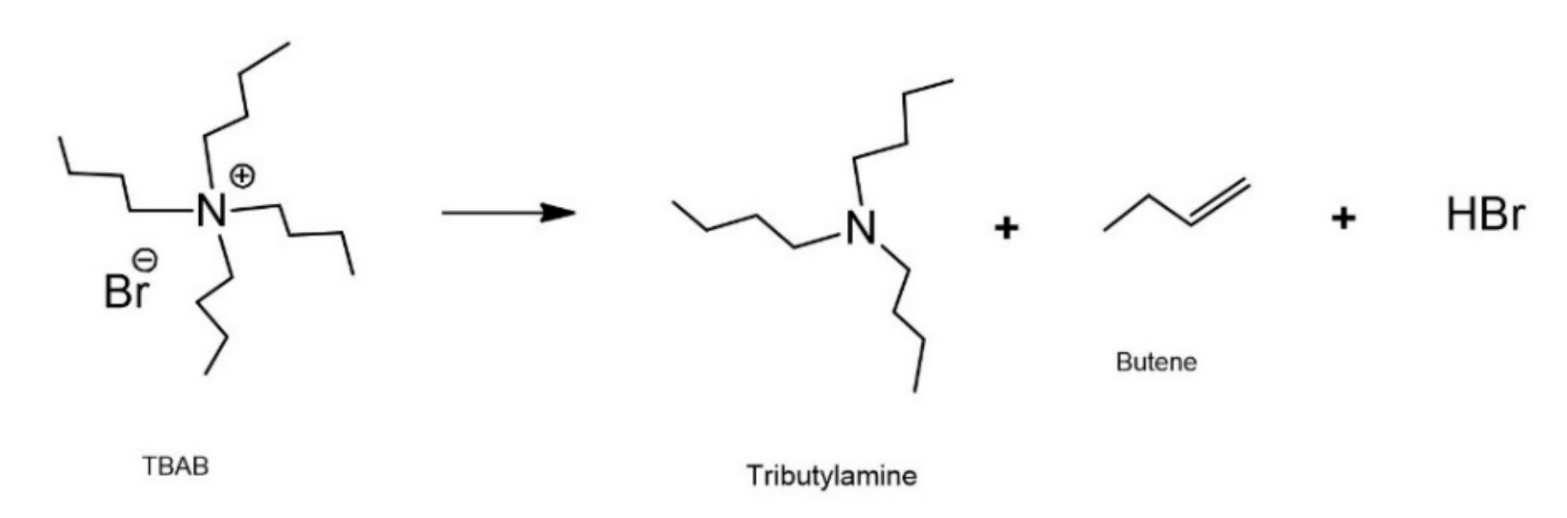
Products obtained from the Hofmann elimination reaction [6].

Although CC has been synthesized since 1933 by Carothers et al. [17], it was not until 2004 that Tamami et al. obtained CC5 from epoxidized soybean oil (ESO) and CO_2_ for the first time in the presence of TBAB as a catalyst (Scheme 1) [18]. A significant amount of research has focused on developing catalysts or co-catalysts to enhance carbonation kinetics. Generally, catalysts are composed of a Lewis acid for the oxirane’s electrophilic activation and a Lewis base that acts as a nucleophile. Some of these catalysts are quaternary phosphate compounds, phosphine complexes, metal complexes, alkali metal halides, quaternary ammonium salts, and ionic liquids, among others [19,20,21,22,23,24,25]. Additionally, it has been observed that the catalytic activity increases when the acidity of the cation and the nucleophilicity of the halide increase [19]. Therefore, co-catalyst systems have been proposed, including CaCl_2_, SiO_2_-I, palladium doped with H_3_PW_12_O_40_/ZrO_2_, TBAB+SnCl_4,_ and TBAB+H_2_O [10,18,19,26]. Among the catalyst systems studied, TBAB is the most commonly used catalyst in the carbonation reaction due to bromine’s effectiveness as a leaving group [27]. However, one disadvantage of TBAB is that at high temperatures (150–190 °C), it shows decomposition to volatile components (Hofmann reaction) (Scheme 2) [6].

Furthermore, several investigations have focused on studying operational and equipment parameters using TBAB as a catalyst. Tamami et al. (2004) carried out the carbonation reaction under mild conditions (atmospheric pressure; 110 °C). Despite obtaining relatively high conversion values (94% by Fourier transform infrared spectroscopy (FT-IR) and 78% by titration) [26], the reaction time was considered long (70 h) and involved an obstacle to commercialization [20]. After that, the studies’ main objective was to optimize the reaction time (Table 1). Javni et al. studied the carbonation of ESBO at higher CO_2_ pressure (56.5 bar), demonstrating that reaction time and conversion are a function of temperature, pressure, and catalyst concentration [2,18]. Doll et al. intensified the carbonation conditions using CO_2_ in the supercritical state (103 MPa, 100 °C) and reduced the reaction time to 40 h and 100% conversion. Similar results were obtained by Mann et al.; however, using supercritical conditions is a disadvantage due to high energy consumption [6,28]. Regarding the efforts to develop equipment to improve carbonation, Mazo et al. used microwave heating (1 atm, 120 °C) and a water ratio (1:3; H_2_O/poxy), reaching 87% conversion in 40 h [29]. Zheng et al. proposed using a continuous-flow microwave reactor, obtaining 73% conversion in 7 h (6 bar, 120 °C). Nevertheless, homogeneity and microwave penetration are still problems to scale to the industry level [30].

Despite the progress made in the investigations of carbonation reactions in vegetable oils, there are still some drawbacks. As shown in Table 1, the main objective of these studies is to achieve high conversion degrees, that is, ensuring that the epoxide groups react mostly. However, the carbonation levels that have been reached are not high, reaching no more than 78% [26,31]. Furthermore, this variable is not measured in most of the reported works. The carbonation level allows us to know if effectively the epoxides are converted into cyclic carbonates or if there is the generation of reaction byproducts that may impact the selectivity of the carbonation reaction.

The conversion kinetics of the reaction improves with increasing temperature and pressure. Nevertheless, the impact of these variables on the degree of carbonation and selectivity has not been studied in detail. Therefore, there are differences in the published results, so it has not been possible to establish the reaction conditions that render a higher percent of carbonation and selectivity, not just conversion (Table 1). This is because we consider it relevant to systematically study the influence of temperature, CO_2_ pressure and TBAB concentration on the conversion, carbonation and selectivity degree to find a balance of those variables for obtaining the highest reaction parameters.

**Table 1:** Literature Reports of Carbonation Reaction in Epoxidized Vegetable Oils (EVOs) Using Tetrabutylammonium Bromide (TBAB).

Oil Type	Catalyst Type	Reaction Conditions (Carbonation)				Reaction Results (Carbonation)			Equipment/ Process Characteristics	Reference
		Pressure	Temperature	Time	% Catalyst	% Conversion	% Carbonation	% Selectivity		
ESO	TBAB	1 atm	120 °C	70 h	5%	87%	77%	89%	1:3 (H_2_O/Epoxy)	[8]
ELO	TBAB	10 bar	140 °C	96 h	---	91%	26.7%	---	---	[18]
ESO	TBAB	1 atm	110 °C	70 h	5%	94%	---	---	Constant CO_2_ flow	[20]
ESO	TBAB	1 atm	110 °C	89 h	2.5%	63%	---	---	---	[2]
		57 bar	140 °C	20 h	2.5%	100%	---	---	---	
ESO	TBAB	10 bar	120 °C	20 h	3%	71.3%	---	---	Autoclave	[28]
ECSO	TBAB	30 bar	140 °C	24 h	3.75%	99.9%	---	---	Autoclave	[32]
ESO	TBAB	103 bar	100 °C	40 h	5%	100%	---	---	Supercritical CO_2_	[7]
ECO	TBAB	5 bar	130 °C	8 h	5%	93.4%	57.7%	61.7%	Oxirane esterification	[33]
ECSO	TBAB	6 bar	120 °C	7 h	8%	73%	---	---	Continuous Flow microwave reactor	[30]
EVNO	TBAB	59 bar	100 °C	46 h	---	95.3%	---	---	Supercritical CO_2_ with stirring (150 rpm)	[34]
ESO	TBAB	1 atm	120 °C	40 h	5%	86.7%	77.4%	88.6%	1:3 (H_2_O/Epoxy) + Microwave	[29]

ESO = Epoxidized soybean oil; ELO = Epoxidized linseed oil; ECSO = Epoxidized cottonseed oil; ECO = Epoxidized castor oil; EVNO = Epoxidized vernonia oil.

## 2. Materials and Methods

### 2.1. Materials

Linseed oil (LO) was purchased commercially through a local distributor (Jalisco, Mexico). LO has a clear yellow oil appearance. The molecular weight (M_W_), the number of double bonds (DB), and iodine value (IV) were determined by proton nuclear magnetic resonance (^1^H-NMR) as described in [9,35,36], rendering 920.5 g/mol, 6.86 (DB), and 189.16 (IV), respectively. IR120 (AIR-120H) Amberlite catalyst (1.8 meq/mL by wetted bed volume), TBAB (≥98.0% assay), solvents such as ethyl acetate (≥99.5% assay) and toluene (≥99.5% assay) were purchased from Sigma-Aldrich Química, S.L. (Toluca, EdoMex, México) Acetic acid (≥99.7%), and hydrogen peroxide (50% concentration) were obtained from Fermont (Monterrey, N.L, México). Industrial grade CO_2_ gas (≥99.5%) was purchased from Praxair (Toluca, EdoMex, México). All reagents were used as received except the LO, which was passed through a chromatographic column filled with α-alumina.

### 2.2. Characterization

The structural analysis of products and the monitoring of epoxidation and carbonation reactions were studied using FT-IR, Carbon-13 nuclear magnetic resonance (^13^C-NMR), and proton magnetic resonance (^1^H-NMR) spectroscopies. FT-IR spectra were obtained on an FT-IR Prestige 21 spectrometer, Shimadzu Scientific Instruments, Inc. in México (Tultitlán, EdoMex, México) equipped with a diamond crystal and a horizontal attenuated total reflectance (HART) module. The infrared spectra were obtained in absorbance mode with 64 scans and a resolution of 4 cm^−1^ in the range of 560–4000 cm^−1^. All FT-IR spectra were normalized to the signal at 1736 cm^−1^ [37], corresponding to the triglyceride ester group’s carbonyl vibration. An Avance III spectrometer (Bruker Mexicana, S.A. de C.V, Cd. de México, México) was used for ^13^C-NMR and ^1^H-NMR analysis. The analysis was performed at 300 MHz, with a spectrum width of 3689.22 Hz, a pulse width of 4.75 µs, 32 scans at 293 K, 90 pulse width of 9.5 µs. CDCl3 was used as the solvent, and tetramethylsilane was used as the internal standard. Through ^1^H-NMR, the number of epoxide and carbonate groups was quantified, as well as conversion, epoxidation, carbonation, and selectivity values of the reactions [8,13,35].

### 2.3. Synthesis of Epoxidized Linseed Oil (ELO)

**Scheme 3:**
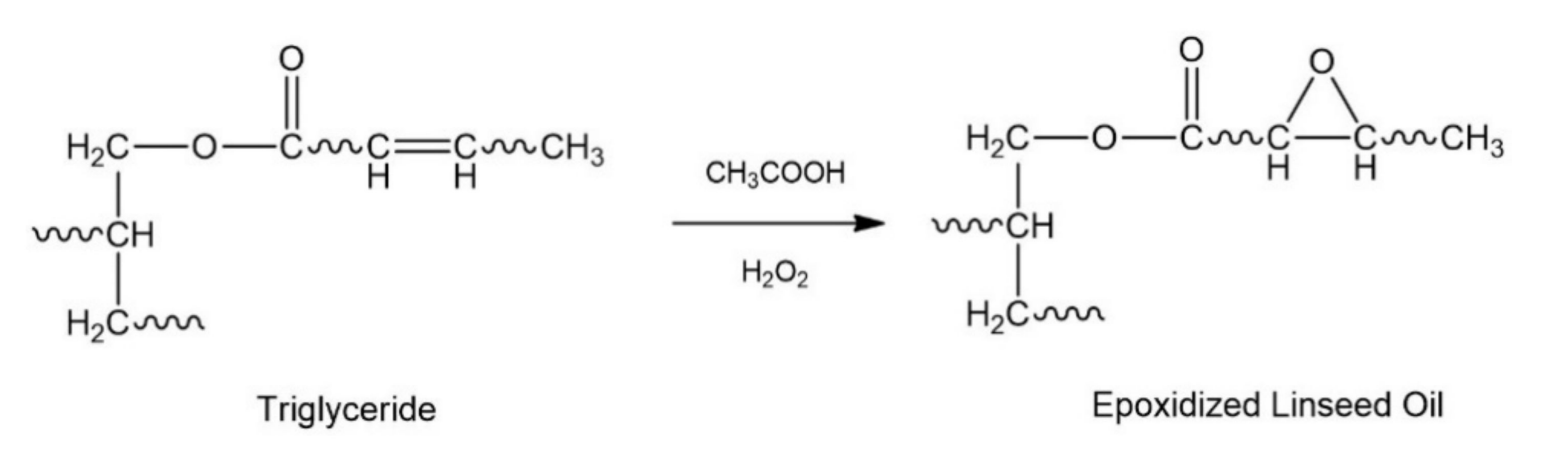
Epoxidation reaction of vegetable oils (VOs).

ELO was obtained according to the Prileschajew reaction (industrial method), where DB of LO reacts in situ with a percarboxylic acid (peracetic acid) in the presence of a heterogeneous catalyst (Scheme 3), such as an ion-exchange resin (Amberlite IR120) [38,39,40]. The conditions of the epoxidation reaction were obtained from a recently optimized methodology [35]. The general procedure consists of placing LO (0.054 mol), toluene (25 mL), acetic acid (0.53 mol/DB), and Amberlite IR120 (12.5 g) inside a three-necked flask equipped with a thermometer, magnetic stirring, and condenser with reflux. The initial mixture was heated to 50 °C, and H_2_O_2_ (50 wt%) (1.54 mol/DB) was added. Subsequently, the reaction temperature was adjusted to 80°C and maintained under these conditions for 90 min. Immediately, the reaction mixture was cooled to room temperature, and the product was purified. Then, 150 mL of ethyl acetate was added to the mixture and filtered under vacuum to remove the catalyst. The organic phase was washed with 500 mL of a 10% sodium bicarbonate solution to neutralize acetic acid. Subsequently, the organic phase was dried with anhydrous magnesium sulfate and filtered. The solvent is removed using a rotary evaporator and then placed in a vacuum desiccator [37]. Finally, the product obtained was characterized by both FT-IR and ^1^H-NMR.

### 2.4. Synthesis of Carbonated Epoxidized Linseed Oil (CELO)

As shown in Table 1, various authors have systematically investigated the effect of different variables, both in the process and in operation, on the epoxidation degree of vegetable oils [8,26,32]. From the results obtained, it has been shown that high epoxidation values can be obtained and are spectroscopically similar to each other (e.g., soybean oil, cottonseed oil, linseed oil). However, in most of the studies, a low carbonation degree was obtained. Therefore, it was necessary to study different reaction conditions to determine the best possible parameters to obtain carbonated oils with the highest carbonation degree and reaction selectivity. The carbonation reaction was monitored as a function of time, temperature, CO_2_ pressure, and catalyst concentration, quantifying by ^1^H-NMR the content of oxirane and carbonate groups to determine the change in the epoxidation, carbonation, and selectivity percentage. The synthesis was carried out in 500 mL Teflon vessel, inserted into a stainless steel reactor equipped with a temperature controller from room temperature to 400 °C and inlets necessary to flow gas and pressurize the reaction in a range from 15 to 200 psi. All the settings of the experimental conditions and the reaction parameters are shown in Table 2. The first variable that was studied was temperature. The reaction temperature was modified from 90 to 120 °C, keeping the pressure (90 psi) and the catalyst concentration (2.5%) constant and setting the temperature that presented the best carbonation reaction results. Subsequently, CO_2_ pressure was modified in an interval from 60 to 120 psi, keeping the temperature (90 °C) and catalyst concentration (3.5%) constant. Finally, once the impact of both the temperature and the CO_2_ pressure on the reaction performance was defined, the catalyst concentration (from 2.5% to 5.0%) was varied at temperature (90 °C) and constant pressure (60 psi). Finally, once the effect of both temperature and CO_2_ pressure on the reaction yield was defined, the catalyst concentration (from 2.5% to 5.0%) was varied at constant temperature (90 °C) and pressure (60 psi). The general methodology consisted of placing 5 g of ELO (M_W_ = 1007.3 g/mol; 6.26 epoxide content, EC, values obtained by ^1^H-NMR) and the amount of TBAB (2.5–5.0% mol with respect to EC) inside the reactor. The mixture was stirred manually for 10 min until complete homogeneity was achieved and the reactor was closed. Prior to initiating the reaction, an oxygen-free atmosphere is generated by performing three CO_2_ purges. Afterward, the system is adjusted to reaction conditions by holding both temperature (90–120 °C) and CO_2_ pressure (60–120 psi) constant for a defined time (12–92 h). After the reaction time was increased, the system was cooled to room temperature and depressurized. ELO purification is initiated by solving the reaction mixture with ethyl acetate. Subsequently, at least 3 or 5 washes with hot water (50 °C) are carried out to remove the catalyst. The organic phase is dried with anhydrous magnesium sulfate. The highest amount of ethyl acetate is removed by distillation at reduced pressure, and the residual solvent is eliminated in a vacuum desiccator for 24 h. Finally, the dry samples were characterized by FT-IR and ^13^C-NMR. The amount of carbonate groups formed was quantified by ^1^H-NMR. The runs were performed in triplicate and the results plotted are the average value of each test.

## 3. Results

### 3.1. Characterization of Epoxidated Linseed Oil (ELO)

**Figure 1:**
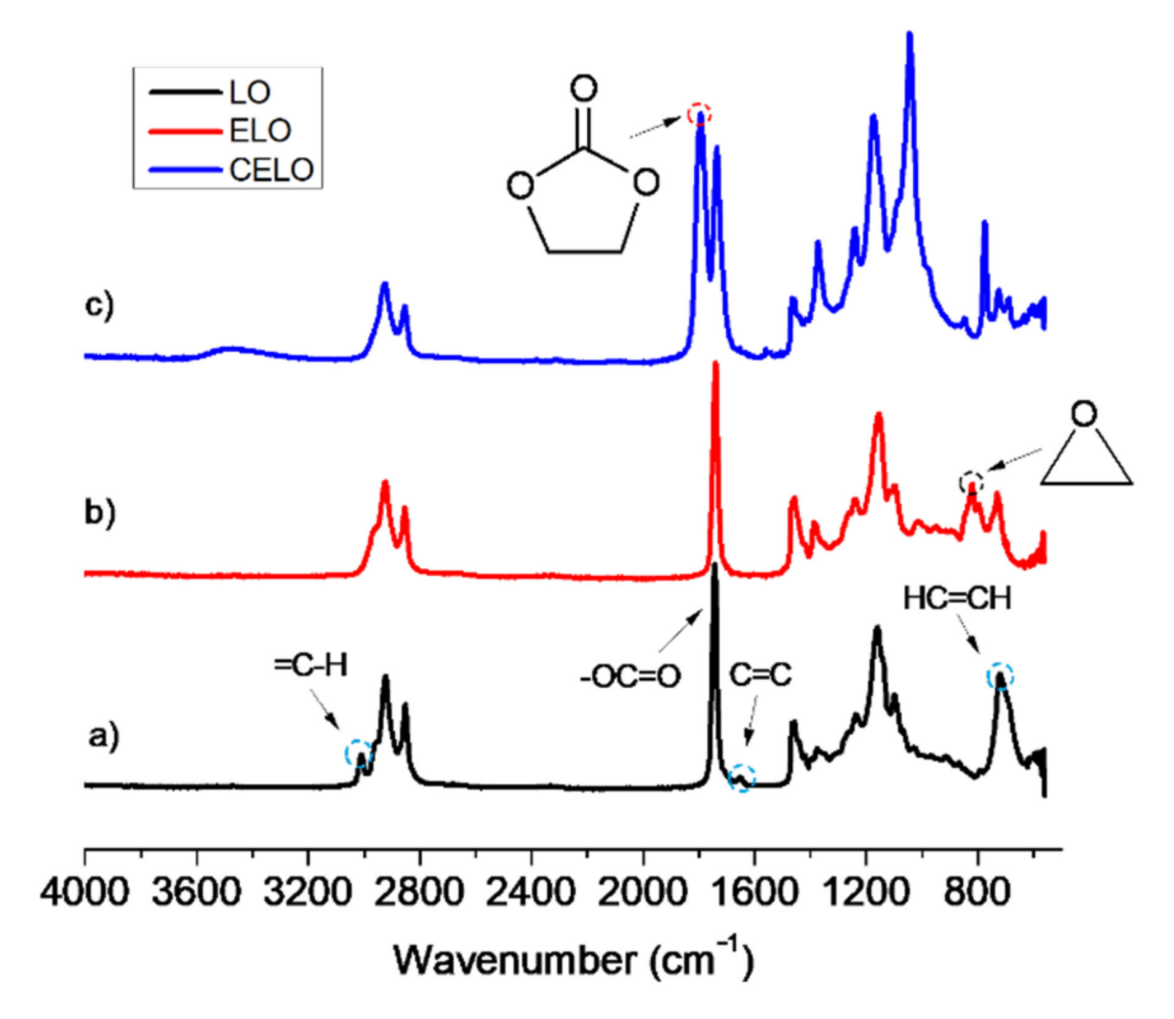
Fourier transform infrared spectroscopy (FT-IR) spectrum of (**a**) linseed oil (LO), (**b**) epoxidized linseed oil (ELO), and (**c**) carbonated epoxidized linseed oil (CELO).

FT-IR spectra were obtained for both the raw material (LO) and the corresponding epoxide (ELO), consistent with those reported by different authors [35,37,41,42,43]. The most representative LO vibration signals (Figure 1a) correspond to the ester carbonyl group at 1736 cm^−1^ (C=O) and double-bound signals at 3021 cm^−1^ (=C–H), 1652 cm^−1^ (C=C) and 720 cm^−1^ (HC=CH_cis_). The other vibration bands correspond to ester carbonyl (1736 and 1159 cm^−1^), methyl (2922, 1456, and 1377 cm^−1^), and methylene (2852 and 719 cm^−1^) groups. In the ELO spectrum (Figure 1b), the epoxy ring’s vibration signals are identified at 1250 and 823 cm^−1^ (C–O–C), the last being the most representative. The FT-IR technique allowed us to qualitatively verify the formation of ELO from LO through the disappearance of DB signals and the appearance of bands corresponding to the epoxy rings. Moreover, it is important to mention that there is no evidence of hydrolyzed chains or interruption of ester bonds, which generally occur at 3200–3550 cm^−1^ (hydroxyl zones) and at 1650 cm^−1^ (carboxylic acids), respectively [37,44].

**Figure 2:**
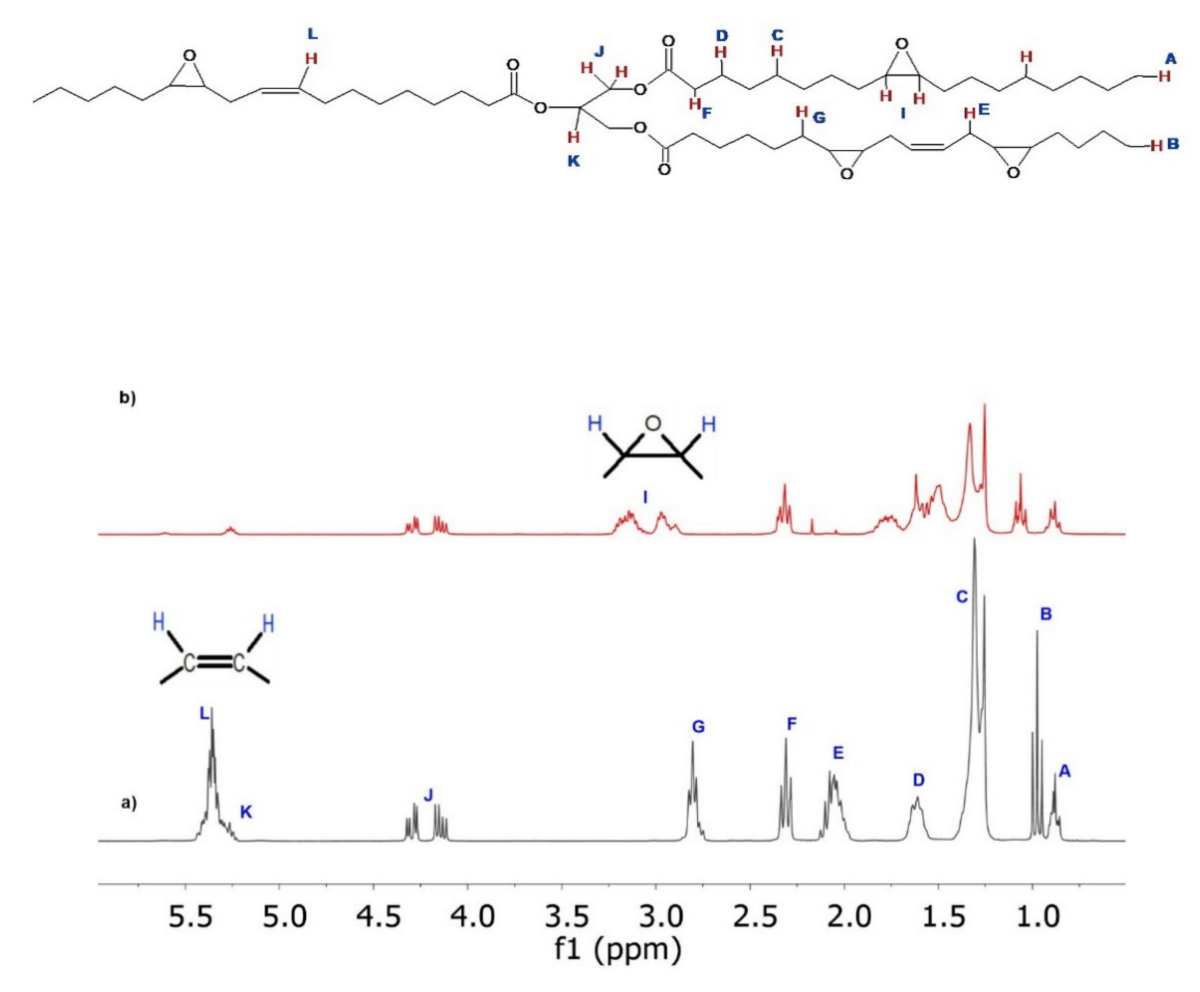
Proton nuclear magnetic resonance (^1^H-NMR) spectra of (**a**) LO and (**b**) ELO.

The signals of representative ^1^H-NMR spectra for LO and ELO are shown in Figure 2, which are consistent with those reported in the literature [35,37,45]. In the ^1^H-NMR spectrum of LO (Figure 2a), the most characteristic signal belongs to DB (vinyl hydrogens) at 5.25–5.45 ppm (L), which overlaps with the central hydrogen of glycerol (K). The rest of the hydrogen signals are found at 4.10–4.34 ppm (J, glycerol methylene), 2.74–2.80 ppm (G, internal allylic), 2.27–2.35 ppm (F, α-carboxylic), 1.97–2.13 ppm (E, external allylic), 1.55–1.68 ppm (D, β-carboxylic), 1.23–1.40 ppm (C, aliphatic methyl), 0.94–1.01 ppm (B, fatty acid methylene) and 0.84–0.92 ppm (A, methyl). The ^1^H-NMR spectrum of ELO (Figure 2b) is similar to that of LO; hence, the presence of an oxirane ring was corroborated mainly with the appearance of the signal in the region of 2.86–3.23 ppm (I, –CHOCH–). Furthermore, a significant decrease and signal shift corresponding to the unreacted vinyl hydrogens (5.6 ppm region) is observed. The epoxide group content present in ELO, as well as the molecular weight of oil, was determined by ^1^H-NMR, giving values of 1007.3 g/mol and 6.26 epoxide groups, respectively [35].

### 3.2. Characterization of Carbonated Epoxidized Linseed Oil (CELO)

CELO was obtained by reacting the oxirane groups of ELO obtained above with CO_2_ in the presence of TBAB. The reaction mechanism that has been adopted involves a nucleophilic attack by the bromide ion on the epoxy ring. Alkoxide generation is promoted, which in turn carries out a nucleophilic attack on CO_2_. Finally, the oxyanion displaces the bromine, generating the corresponding CC5 (Scheme 1) [8,18].

The structural characterization of CELO was carried out using FT-IR, ^13^C-NMR, and ^1^H-NMR spectroscopic techniques that are consistent with the results reported in the literature [6,17,20,31,46,47,48]. To corroborate the formation of the carbonate group, the infrared spectra of ELSO and CELO were compared. In the CELO spectrum (Figure 1c), the carbonate group’s vibration signals appear at 1798 and 1045 cm^−1^. The most notable signal is at 1798 cm^−1^, which corresponds to the carbonyl of the cyclic carbonate (C=O), while the signal at 1045 cm^−1^ is assigned to the C-O bond. Also, the disappearance of epoxide group signals (823 cm^−1^) is observed.

**Figure 3:**
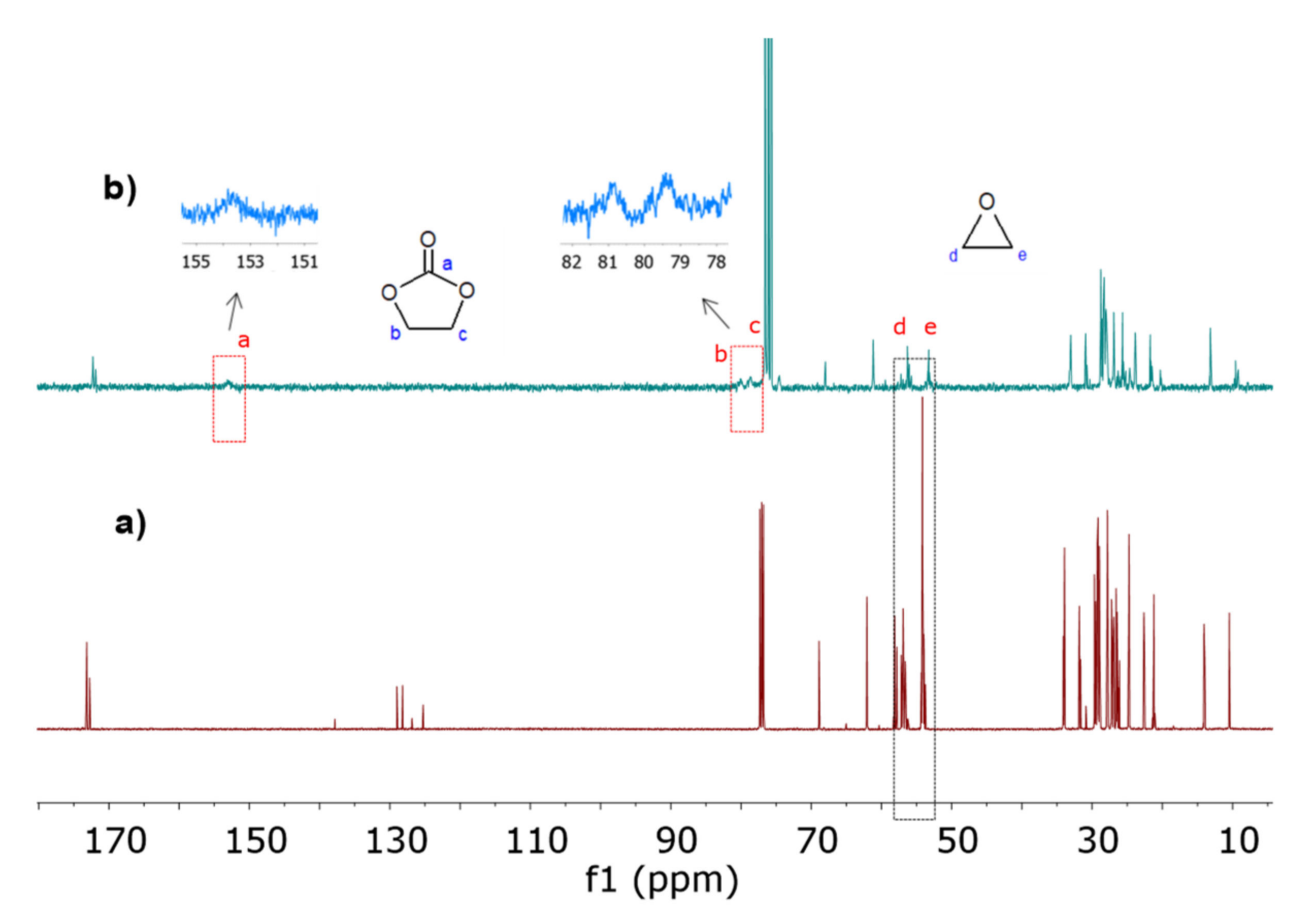
Carbon-13 nuclear magnetic resonance (^13^C-NMR) spectra of (**a**) ELO and (**b**) CELO.

As additional qualitative evidence, ^13^C-NMR spectra were useful to identify the carbonate group’s presence in the CELO structure, as reported by Doley and Dolui, Zheng et al. and Liu and Lu [10,47,48]. The ^13^C-NMR spectrum of CELO (Figure 3b) showed the following carbon signals: end methyl (9.7–13.3 ppm), triglyceride chain methylene (20.2–33.4 ppm), remaining epoxy rings (53.2–57.4 ppm), central methine (61.5 ppm), methylene of glycerol (68.2 ppm), and carbonyls of fatty acids (172.5–173.0 ppm). Carbon signals at 79.2 ppm (C–O), 81.4 ppm (C–O), and 154.1 ppm (C=O) confirm the formation of CELO.

**Figure 4:**
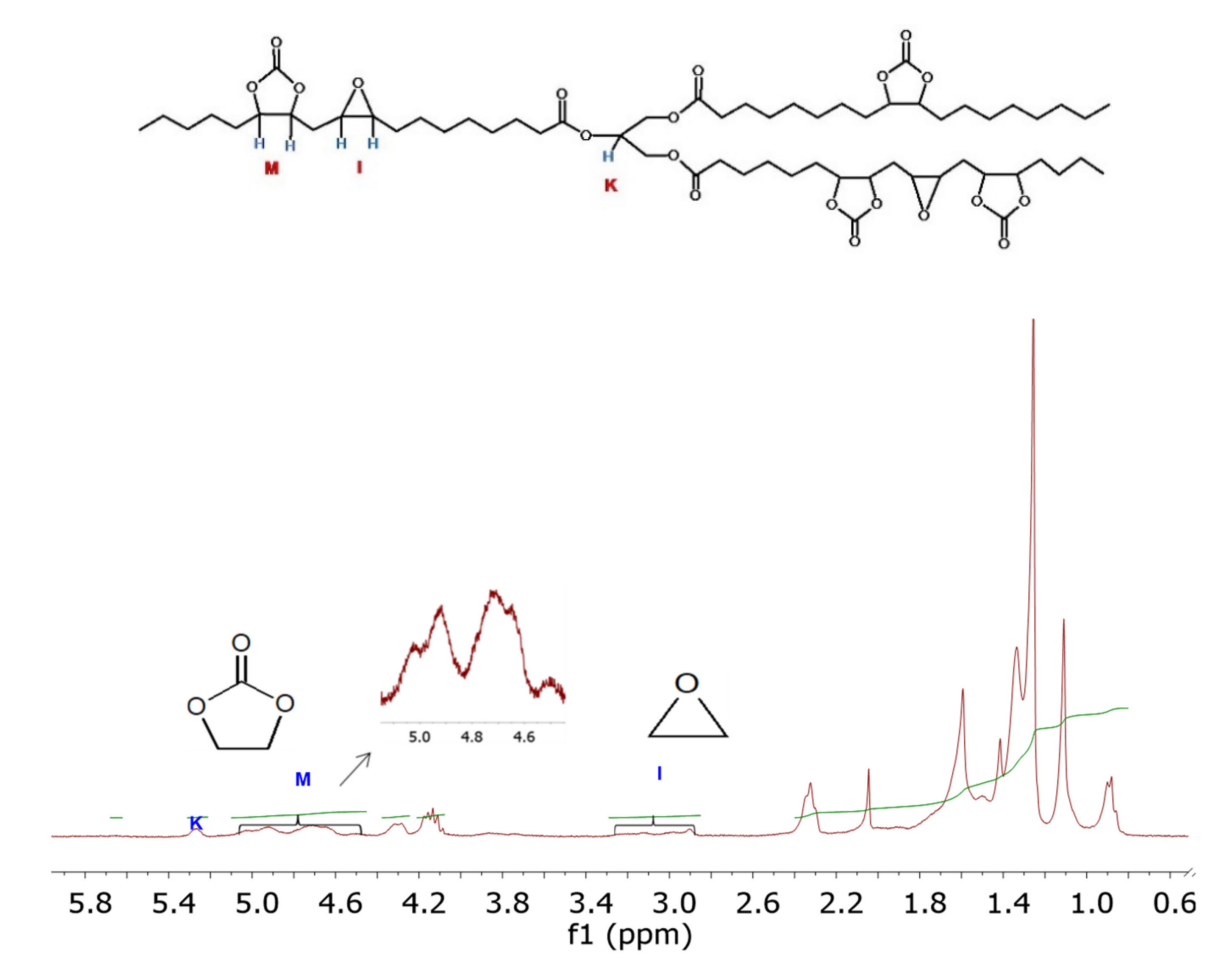
Values obtained by ^1^H-NMR in the carbonation reaction at 90 °C, 60 psi, 3.5% TBAB, and 68 h.

Figure 4 shows a representative ^1^H-NMR spectrum of CELO, which was useful for qualitative characterization of CELO and quantifying the carbonation reaction progress. In the ^1^H-NMR spectrum of CELO (Figure 4), the signal corresponding to the hydrogen of the central carbon of glycerol at 5.25 ppm (K) is observed. In the 2.85–3.28 ppm (I) region, the hydrogen is associated with the unreacted epoxide groups. To corroborate CELO formation, signals associated with cyclic carbonate hydrogens appear in the interval of 4.45–5.10 ppm (M).

The ^1^H-NMR technique is widely used to characterize materials’ chemical structure, both qualitatively and quantitatively [49]. ^1^H-NMR has been shown to be an effective technique, compared to traditional methods, to determine the chemical composition of triglycerides and their derivatives and reaction parameters [36,50]. The performance of the carbonation reaction was evaluated through conversion (% C), carbonation, or yield (% Y), and selectivity (% S) parameters. The numbers of epoxide and carbonate groups present in CELO were calculated from the integrals of the ^1^H-NMR spectrum (Figure 4). The central carbon (K) signal was taken as the spectrum normalization factor. The number of epoxide groups (E_m_) was calculated with the signals corresponding to epoxy rings (I) (Equation (1)). Similarly, through Equation (2), the number of carbonate groups (C_m_) was calculated from the associated signals (M). Regarding the reaction parameters, the conversion, carbonation, and selectivity values were obtained using Equations (3)–(5) [8,29]. The runs were performed in triplicate and the results plotted are the average value of each test:

(1) $$E_{m}=\frac{I}{2K}$$

(2) $$C_{m}=\frac{M}{2K}$$

(3) $$\%C=\left(\frac{E_{\mathrm{mi}}-E_{\mathrm{mf}}}{E_{\mathrm{mi}}}\right)\times 100$$

(4) $$\%Y=\left(\frac{C_{m}}{E_{\mathrm{mi}}}\right)\times 100$$

(5) $$\%S=\left(\frac{\%Y}{\%C}\right)\times 100$$

#### 3.2.1. Effect of Temperature

To determine the optimal conditions for obtaining CELO that contains the highest degree of carbonation (high content of carbonate groups) and selectivity (least amount of byproducts), the carbonation reaction was monitored based on temperature, CO_2_ pressure, and the amount of catalyst. In general, in the CELO structure, the epoxide groups show steric hindrance because the epoxides’ positions are located in the middle of the molecular chain (25). To obtain high levels of conversion, it is necessary to use a slightly elevated temperature (Scheme 2), avoiding reaching the decomposition temperature of the catalyst (determined as 150–190 °C by Doll et al. [6]). Therefore, temperature is a critical parameter in the carbonation reaction.

**Figure 5:**
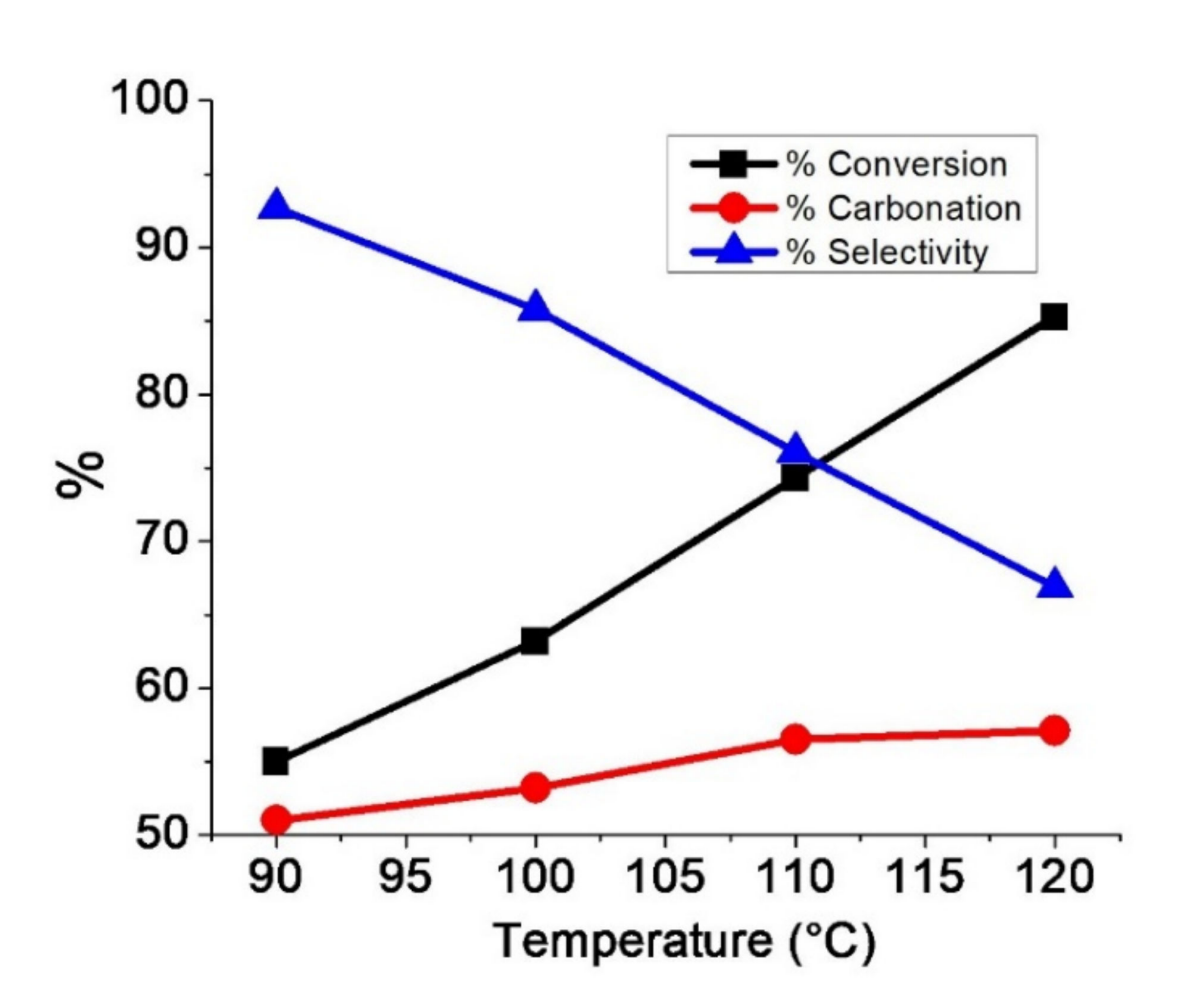
Effect of temperature on the conversion, carbonation, and selectivity degree.

Figure 5 shows the effect of reaction temperatures (90, 100, 110, and 120 °C) on the conversion, carbonation, and selectivity degree while keeping the pressure (90 psi), catalyst concentration (2.5% TBAB), and time (24 h) constant. At 90 °C, conversion reaches 55%. When the temperature increases from 90 to 120 °C, the conversion increases from 55 to 85.3%. Similar behavior is observed in the carbonation percentage. By increasing the temperature to 120 °C, carbonate formation increases slightly from 51 to 57.1%. It is observed that the growth rate in carbonation is slower than that in conversion. The opposite behavior is observed in selectivity, whereby increasing the temperature to 120 °C, the selectivity decreases from 92.7 to 66.9%. Through FT-IR, it was detected that as the reaction temperature increased from 90 to 120 °C, the signal corresponding to the hydroxyl zone also increased (3200–3600 cm^−1^). Usually, in chemical reactions, it is desirable to have high selectivity values. Low values indicate that some of the epoxide groups are not being converted to the desired product, i.e., they are not being formed to cyclic carbonates, which indicates that some alternative reactions.

**Figure 6:**
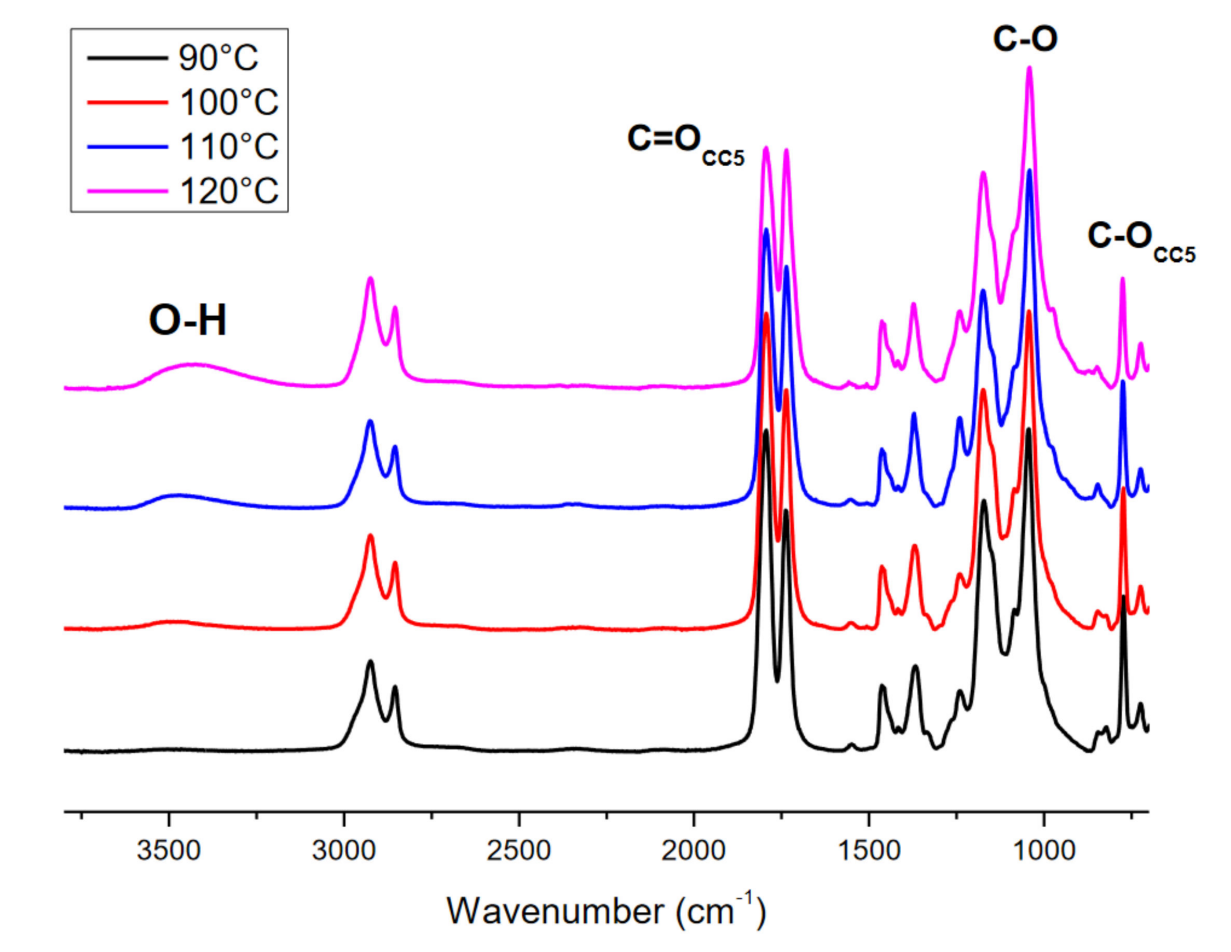
FT-IR spectrum of CELO at different temperatures.

**Scheme 4:**
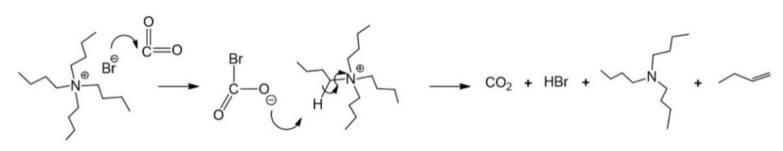
Byproducts resulting from the interaction between TBAB and CO_2_ at 120 °C.

**Figure 7:**
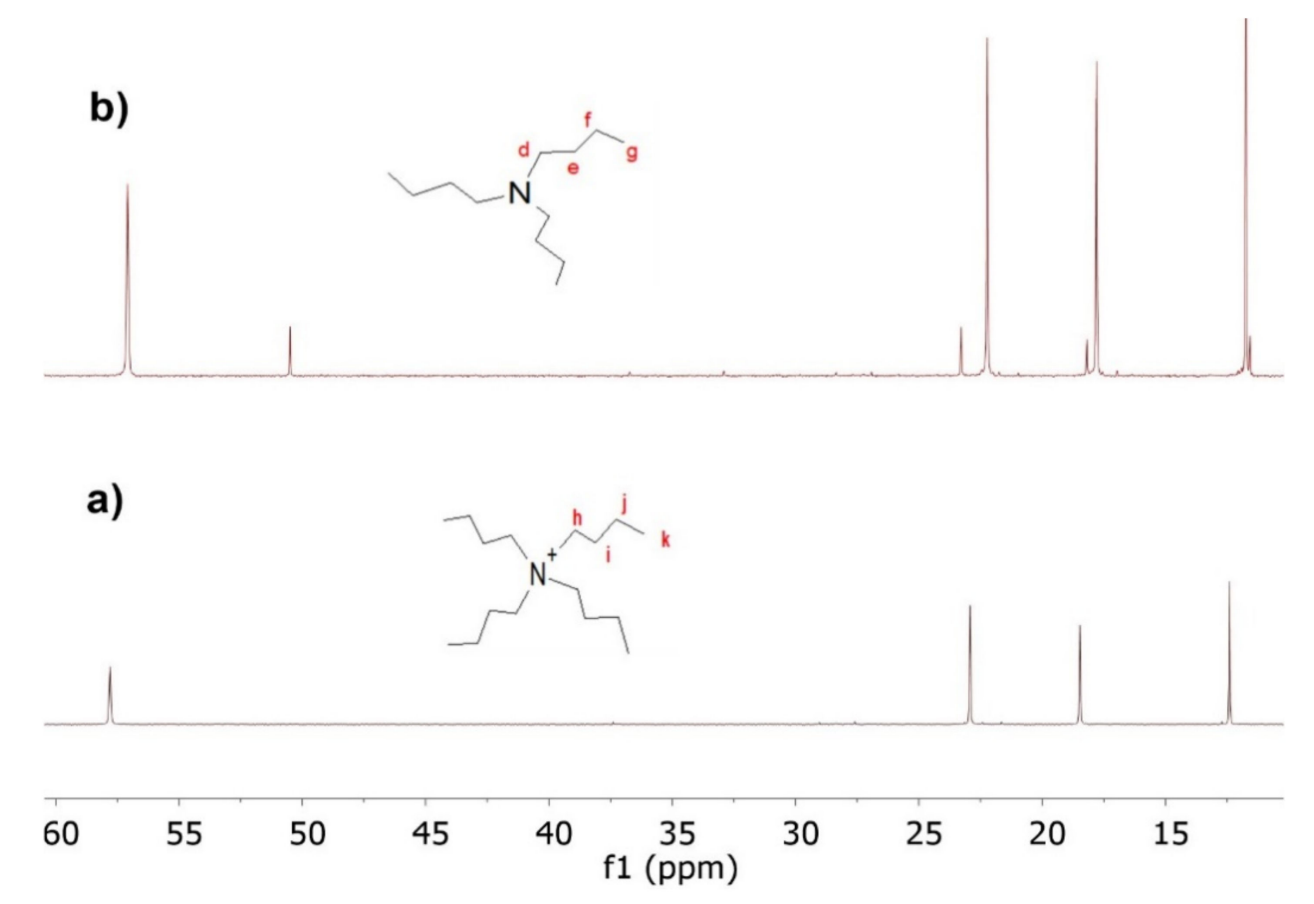
^13^C-NMR spectrum of (**a**) TBAB, and (**b**) trimethylamine as a byproduct or the TBAB+CO_2_ reaction in absence of ELO at 120 °C, 90 psi, and 48 h.

Although the carbonation reaction was carried out below the decomposition temperature of TBAB, evidence of the formation of byproducts at higher temperatures is presented in Figure 6. To verify this statement, the interaction between TBAB and CO_2_ was studied in the absence of ELO at 120 °C and 90 psi for 48 h. TBAB decomposition is similar to the Hoffman reaction (Scheme 3). However, due to the presence of CO_2_, the first step was the addition of the bromide to the CO_2_ molecule to form the carboxylate ion, which extracts beta hydrogen from a butyl substitute of tetrabutylammonium, decomposing into CO_2_ and hydrogen bromide, and generating butene and tributylamine as final products (Scheme 4). Figure 7 shows the ^13^C-NMR spectra of the catalyst before (pure TBAB) and after being subjected to heat treatment in the presence of CO_2_ (Figure 7b). The carbon signals of pure TBAB (Figure 7a) are found at 12.2 ppm (*d*, terminal methyl), 18.5 ppm (*e*, methylene), 22.8 ppm (*f*, methylene), and 57.8 ppm (*g*, quaternary amine). In Figure 7b, the formation of tributylamine is confirmed by the appearance of signals at 11.5 ppm (*h*, –CH3), 18.2 ppm (*i*, –CH2–), 23.2 ppm (*j*, –CH2–), and 50.4 ppm (*k*, tertiary amine). Because the spectrum was only obtained from the solid sample, the presence of butene and carboxylate was determined indirectly. However, it is necessary to continue with the byproducts characterization.

**Scheme 5:**
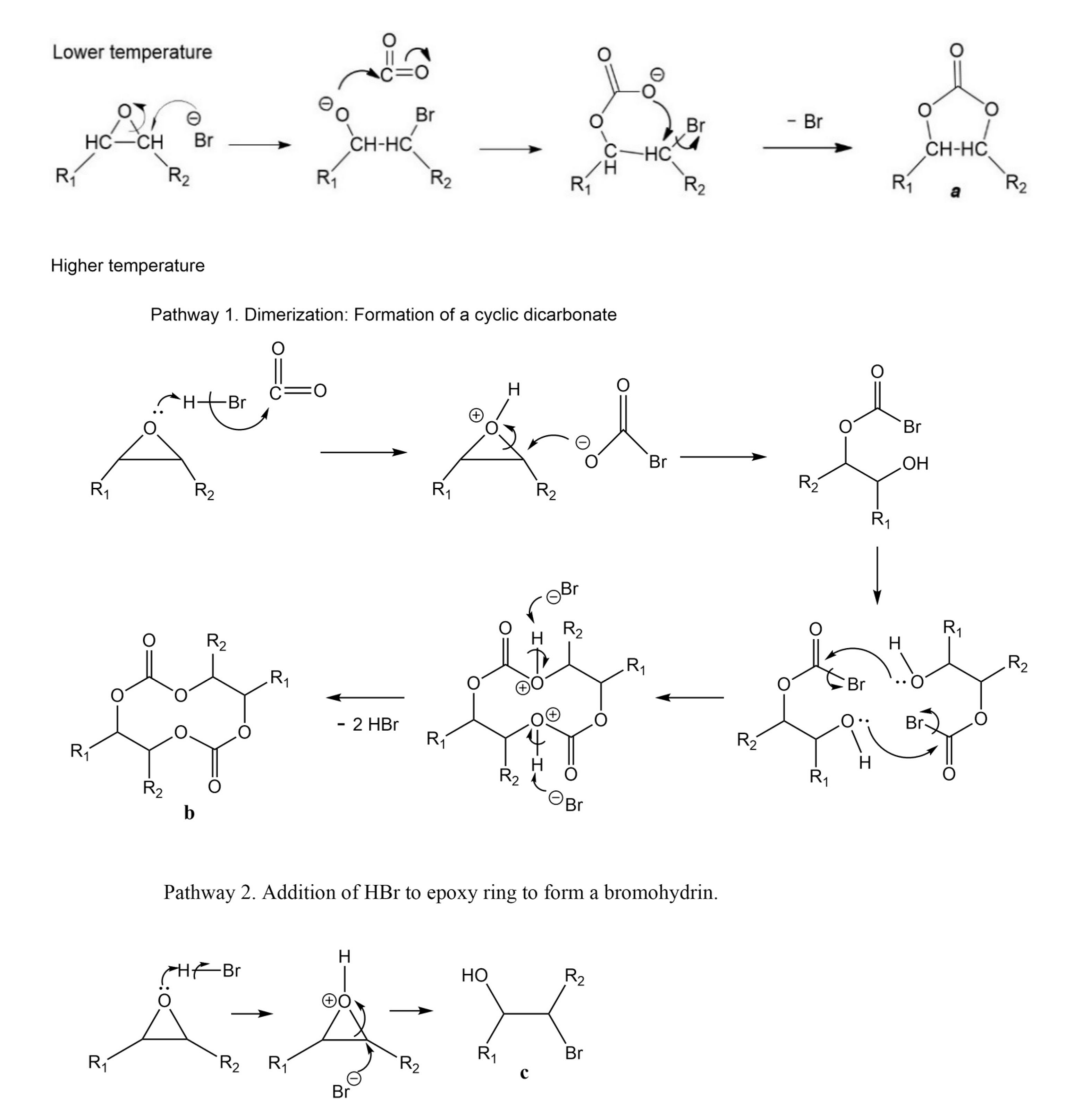
Possible reaction pathways between CO_2_ and epoxides at low and high temperatures.

The formation of carboxylate ions from metal catalysts and CO_2_ at elevated pressures is well known [51]. The presence of another additional reaction mechanism to the one reported (Scheme 1) is possible at elevated temperatures and in the presence of the carboxylate ion. Hence, the interaction between epoxide and CO_2_ may also depend on the reaction temperature. Similar results were reported by Kiara et al., 1993, who proposed the influence of CO_2_ pressure on the reaction mechanisms [52]. At a lower temperature, the bromide ion generates the alkoxide that is subsequently transformed into the corresponding cycle (species 5a). However, when the carbonation reaction is carried out at a higher temperature, there may also be the formation of other species (oligomers) that negatively impact selectivity. A possible mechanism is shown in Scheme 5 (pathway 1). HBr promotes the formation of carboxylate ions, which by nucleophilic addition, interact with epoxide cations to form carboxylates. However, this species is not stable, so it reacts with another carboxylate molecule to form the final macrocyclic dimers (species 5b) or oligomer. However, analyzing closely the spectra in Figure 6 for runs 1 through 4 (Table 2), new bands corresponding to O–H bonds in 2200–3600 cm^−1^ region were observed for experiments at 100, 110 and 120 °C, as well as a broadening of the band from 990–1070 cm^−1^ due to the overlap of new C–O bonds. This broadening was determined from the area under the curve in the 990–1122 cm^−1^ region, from the normalized spectrum. These facts suggest that formation of hydroxyl groups. One explanation is that HBr generated at temperatures above 100 ° C carried out an epoxy ring opening reaction by the mechanism shown in Scheme 5 (pathway 2) forming a bromohydrin, instead of a CC5 (a) or a dicarbonate (c). Table 3 summarizes signals observed in the ^13^C– and ^1^H-NMR spectra for run 4 (120 °C), which would correspond to a bromohydrin (c). These assignments are supported by the work of Eren et al. [53]. These signals confirm that an increase in temperature enables the epoxy rings to react in alternative ways, decreasing the selectivity towards the carbonation reaction.

#### 3.2.2. Effect of Pressure and Catalyst Concentration

**Figure 8:**
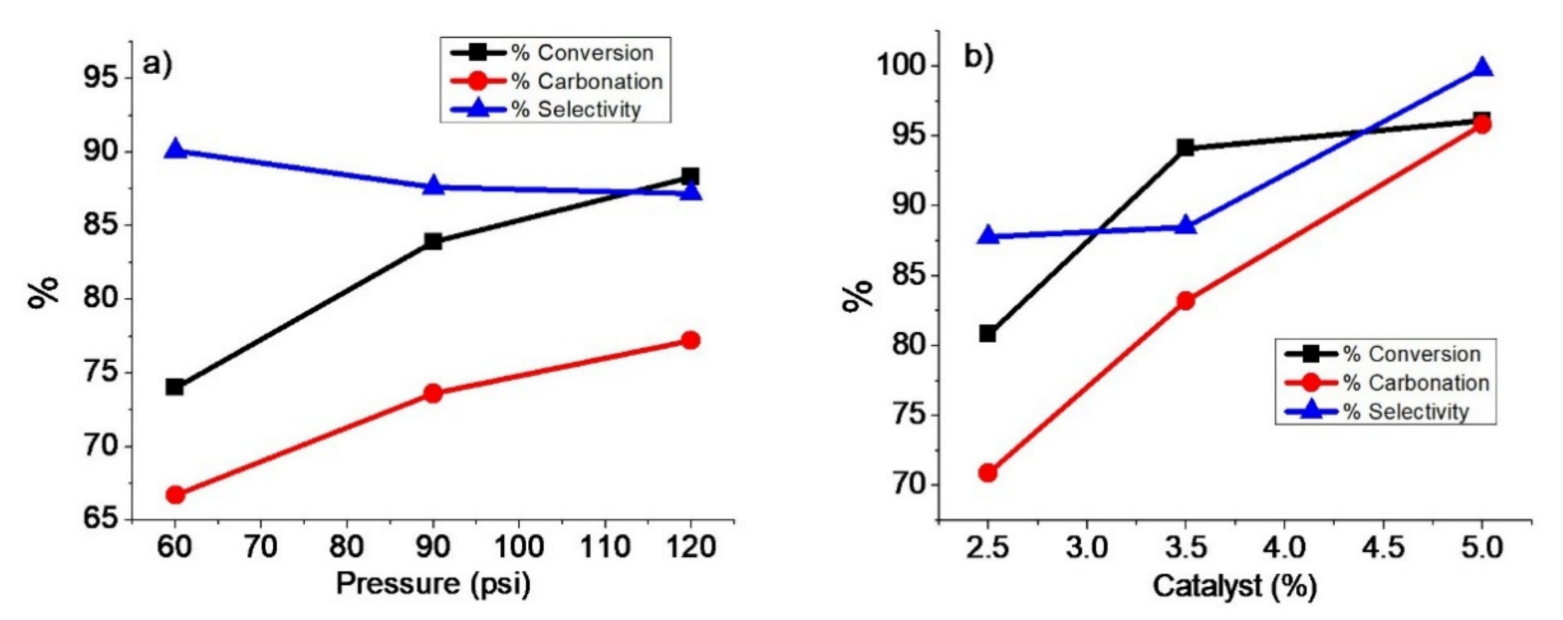
(**a**) Effect of pressure at 90 °C, 3.5% TBAB, and 68 h. (**b**) Effect of catalyst concentration at 120 psi, 90 °C, and 86 h.

Like temperature, pressure is a key parameter in the carbonation reaction, not only because it increases the solubility of CO_2_ in oils but it also favors the carbonation reaction since it is the volume reduction reaction and it promotes the interaction between the oil and the catalyst as observed and studied by other authors [28,32,54,55]. Their studies show that the solubility of a gas (CO_2_) in the liquid phase (ELO) is a function of the pressure of the system and is related by the Henry coefficient. In turn, this coefficient depends on both the properties of the liquid and the temperature [55]. Results published by Zhang et al. [32] have shown that the solubility of CO_2_ increases with pressure, which can favor the conversion of epoxidized vegetable oil (opening of the oxirane ring) due to the higher concentration of CO_2_ in the system. Regarding the glyceride series, they showed that the difference in polarity and molecular weight of the tested molecules affects the solubility in CO_2_. Monoglyceride is logically more soluble in CO_2_ than diglyceride and triglyceride, due to its lower number of carbons, evidence that the solubility of these derivates in carbon dioxide is governed mainly by their molecular weight. A terminal epoxy fatty acid diester was found to be more soluble and more reactive in CO_2_ than an internal epoxy fatty acid diester [56,57]. The reactivity centers, i.e., epoxide group of epoxidized fatty esters is more accessible than the ones of epoxidized vegetable oils. Considering temperature and pressure, the higher they are, usually the greater the solubility of the epoxy ester in CO_2_. They explain this effect by the fact that the increase in temperature leads to a decrease in the cohesiveness of the oil and, therefore, to an increase in its solubility in CO_2_. Furthermore, due to the high molecular weight, the steric hindrance of ELO structure with limitedly accessible internal epoxy groups; it is believed that pressure and temperature can play an important role in increasing yield and selectivity in this particular epoxidized triglyceride. Therefore, in order to increase the degree of carbonation of ELO, it was established to evaluate the carbonation reaction at 90 °C, 3.5% TBAB, 68 h, and under different moderate pressures of CO_2_ (60, 90 and 120 psi). The results obtained from the reaction parameters (% V, % C and% S) are presented in Figure 8a. A considerable increase in conversion from 60 to 120 psi is observed (74% and 88.3%, respectively). Cyclic carbonate formation is also enhanced with increasing CO_2_ pressure, reaching a maximum carbonation degree of 77.2% at 120 psi. However, the growth rate of carbonation is less than the conversion rate. Hence, the selectivity of the reaction decreases from 90.1 to 87.2% with increasing CO_2_ pressure. According to Ochiai and Endo, the mechanism in reactions between oxirane rings and CO_2_ depends on the system pressure [46]. Therefore, at high pressures, the generation of carboxylate ions is favored, reducing the reaction’s selectivity.

Finally, the concentration of catalyst in the reaction parameters was studied. The experiment was performed at 90 °C, 120 psi, and 86 h. The results are shown in Figure 8b. The conversion degrees achieved were 80.8, 94.1, and 96.1% at 2.5, 3.5, and 5.0% TBAB, respectively. The increase in conversion using 2.5 to 3.5% TBAB is greater than 5.0% because as the reaction progresses, the material’s viscosity is higher, decreasing the absorption of CO_2_. The carbonation degree improves from 70.9 to 95.2% using 2.5–5.0% TBAB, indicating a better interaction between the catalyst, CO_2_, and ELO at 5.0%. Similar behavior occurs with selectivity because as the concentration of TBAB increases, selectivity increases, obtaining maximum values of 99%.

The systematic study of the reaction variables made it possible to find the optimal conditions to obtain CELO with a high content of cyclic carbonates and a low content of residual epoxide groups. The temperature was found to be the most critical variable in the reaction. The selectivity value was lower with increasing reaction temperature. In the literature (Table 1), the carbonation reaction was carried out at 120–140 °C to accelerate it. However, they fail to achieve high carbonation percentages. Therefore, in our work, the temperature of 90 °C was determined as the optimum temperature value because at this temperature, the highest selectivity percentage was obtained. Subsequently, the CO_2_ pressure was modified, and when the pressure increased, both the conversion and carbonation percentage increased. Although the selectivity decreases slightly as the pressure increases, the pressure at 120 psi has a higher reaction rate than that at 60 psi. Hence, 120 psi was considered acceptable and was selected as the optimum pressure value. Finally, at 90 °C and 120 psi, the catalyst concentration was changed from 2.5 to 5.0%. It was observed that as the catalyst concentration increased, both the conversion, carbonation, and selectivity percentage increased considerably. Therefore, 5% was set as the optimal catalyst concentration value because values of 96.1, 95.2, and 99% conversion, carbonation, and selectivity, respectively, were obtained. Therefore, 5% was set as the optimal catalyst concentration value because, at this concentration, the highest conversion (96.1%), carbonation (95.2%), and selectivity (99%) values were obtained. Although conversion values equal to 100% have been obtained in the literature, carbonation values above 77% and selectivity values above 89% have not been reported until now. Obtaining vegetable oils with a high carbonate content is essential to improve the quality of subsequent polymers such as NIPU with the required properties.

## 4. Conclusions

The carbonation reaction between epoxidized linseed oil and CO_2_ in the presence of TBAB as a catalyst was studied by modifying the parameters of temperature (90–120 °C), CO_2_ pressure (60–120 psi), and amount of catalyst (2.5–5.0% TBAB). The presence of CELO was corroborated using FT-IR, ^13^C-NMR, and ^1^H-NMR techniques by the appearance of signals corresponding to cyclic carbonates and a decrease in signals belonging to epoxide groups. The different reaction conditions have a significant impact on the conversion, carbonation, and selectivity degree. It was observed that by increasing the reaction temperature from 90 to 120 °C, both the conversion rate and the carbonation percentage are favored; however, selectivity is negatively impacted. An additional reaction mechanism is promoted, based on the generation of carboxyl ions as the result of the interaction between CO_2_ and TBAB. Pressure exhibits similar behavior, and high CO_2_ pressures (120 psi) also favor side reactions. Contrary to the catalyst concentration, selectivity is favored by increasing the percentage of TBAB in the system. From quantification of the ^1^H-NMR spectra, it was established that at 90 °C, 60 psi, and 5.0% catalyst, it is possible to obtain the highest values reported up to the moment of conversion equal to 96%, 95% carbonation, and selectivity values of 99%.

## Figures and Tables

**Table 2:** Experimental Settings and Average Values of the Reaction Parameters: Conversion, Carbonation and Selectivity Percentages.

Run	Temperature (°C)	Pressure (psi)	Catalyst (%)	Time (h)	Conversion (%)	Carbonation (%)	Selectivity (%)
1	90	90	2.5	24	55.0 ± 0.51	51	92.7
2	100	90	2.5	24	63.2 ± 0.40	53.2	85.8
3	110	90	2.5	24	74.3 ± 0.48	56.5	76.1
4	120	90	2.5	24	85.3 ± 0.59	57.1	66.9
5	90	60	3.5	68	74.0 ± 0.59	66.7	90.1
6	90	90	3.5	68	83.9 ± 0.55	73.6	87.6
7	90	120	3.5	68	88.3 ± 0.61	77.2	87.2
8	90	120	2.5	86	80.8 ± 0.58	70.9	87.8
9	90	120	3.5	86	94.1 ± 0.51	83.2	88.5
10	90	120	5.0	86	96.1 ± 0.56	95.8	99.8

**Table 3:** Signals that Corroborate the Formation of a Bromohydrin as a Side Product of the Carbonation Reaction of ELO (Run 4, 120 °C).

NMR Spectra	Chemical Bond	Signals (ppm)	
		Eren et al., 2004 [53]	Experimental
^13^C-NMR	–CHBr–	64.8	64.4
	–CHOH–	75.3	74.5
^1^H-NMR	–CHBr–	4.0	4.2
	–CHOH–	3.4	3.7

## Data Availability

The data is not yet publicly available as it is necessary to first present the dissertation paper. Once submitted, the data is made public in the Institutional Repository of the Universidad Autónoma del Estado de México (http://ri.uaemex.mx/ accessed on 1 March 2021).
